# Remedies and Their Uses

**Published:** 1907-06-08

**Authors:** 


### Therapeutics.

### Remedies and their Uses.

### The Newer Iodine Compounds.

Ever since the introduction of the iodides various attempts have been made to overcome the untoward results which occasionally attend their administration. Iodism, with all its attendant disagreeable symptoms, is often dreaded by the physician who prescribes the iodides to a patient for the first time. By adding ammonia in the form of the carbonate, or by introducing spt. ammon. arom. into the mixture, iodism may to a large extent be prevented, although it can hardly be avoided altogether. Sometimes by increasing the dose of the iodide the symptoms, curiously enough, disappear. In view of all this, several new iodine compounds have been prepared within recent years, and for all of these it is claimed that they are less liable to derange the stomach and to produce iodism than the ordinary iodides of the Pharmacopœia.

*Iodipin.*—This was first brought before the notice of the profession in 1898. It is a chemical combination of iodine and sesame oil. It is prepared in two strengths. Ten per cent. iodipin contains 10 per cent. of iodine, and 25 per cent. iodipin contains 25 per cent. of iodine. The first is a bright yellow, oily liquid, resembling closely sesame oil in odour and taste, whereas the 25 per cent. strength is an oily, viscous fluid which at low temperatures becomes thick, and almost like treacle. The weaker solution is intended for oral administration, the stronger one is almost solely used subcutaneously. Iodipin is only disintegrated in the intestinal canal, and its absorption takes place entirely from the intestines. The greater portion of iodipin when taken is at once oxidised, and on its oxidation all the iodine separates out as iodide. Excretion is much slower than in the case of the ordinary iodides. Toxic symptoms are less marked, owing to the slow oxidation and protracted elimination of the drug. Symptoms of gastro-intestinal irritation are practically never experienced.

*Therapeutic Applications.*—The indications for the use of iodipin are the same as for the iodides. Thus it finds its chief employment in cases of tertiary syphilis. In such cases the subcutaneous administration is worthy of trial, as by this means we have a continuous liberation of iodine going on, and also we secure that in the oxidation of iodipin the iodine is set free in a nascent state. From the site of injection the iodipin is absorbed very slowly, but is distributed gradually through the system. In this way small quantities of the drug are brought constantly into the circulation. The iodipin should first of all be warmed to the body temperature, so as to render it more limpid. The syringe employed should possess a capacity of at least 10 c.c., and should have a needle of somewhat wide calibre. The injections may be made in the upper arm, between the scapulæ, or in the gluteal region. No evil results ever follow the hypodermic use of iodipin. In most cases 20 c.c. of the 25 per cent. strength may be injected daily. These injections are well tolerated, occasion little or no iodism, and produce a prompt and markedly beneficial effect.

Another class of cases which may be benefited by the administration of iodipin is that of circulatory derangements, such as arterio-sclerosis and aneurism. Here we may give by the mouth from a half to 1 drachm of 10 per cent. iodipin thrice daily, increasing the dose gradually if desired. It is best given in warm milk, or, if preferred, may be administered in gelatine capsules. Then, again, iodipin is of great service in chronic respiratory diseases, such as chronic bronchitis, asthma, pulmonary fibrosis, and emphysema; but it should be given with great care in cases of pulmonary tuberculosis. It has also proved beneficial in chronic rheumatism and other rheumatic affections, such as sciatica. In diseases of the nervous system, such as locomotor ataxy and peripheral neuritis, we have seen good results follow its persistent use. No doubt other uses will suggest themselves, but the conditions already mentioned are those in which we have obtained definite results.

*Sajodin.*—More recently still, Fischer and v. Mering have discovered a further group of iodine compounds which are very readily absorbed, effectively digested, and free from taste. Of these sajodin is the most valuable. It is the calcium salt of a compound iodo-acid. It is, unlike iodipin, in the form of a powder, which is stated to contain 26 per cent. of iodine and 4.1 per cent. of calcium. It is completely insoluble in water. Its uses are similar to those of iodipin. It is of special value in the tertiary manifestations of syphilis, and more particularly in cases presenting gummata in various localities and in affections of the bones. In chronic pulmonary and bronchial diseases, as well as in arterio-sclerosis and aneurism, sajodin is worthy of careful and extended trial.

*Dose and Mode of Administration.*—The initial dose for adults is 10 grains, thrice daily after meals. This may be increased, just like iodide of sodium or potassium, up to 60 or more grains. It is best given dry on the tongue or in wafer paper, and a small glassful of water taken immediately afterwards. It should never be given in the form of compressed tablets. Its tastelessness and freedom from odour make sajodin very acceptable to fastidious patients who cannot take iodipin on account of its distinctly oily taste.

Our experience with iodipin and sajodin proves them to be greatly superior to the iodides in common use. It cannot be said that they do not produce symptoms of iodism, for they undoubtedly do, even when given in small doses well diluted; but the symptoms are never so marked as when the iodide of sodium or of potassium is administered. The special value of iodipin lies in the fact that it may be given hypodermically—a matter of considerable importance when we wish the patient, as, for instance, in aneurism, to be brought rapidly under the influence of the drug.

